# A predictive model of Parkinsonian brain aging based on brain imaging features

**DOI:** 10.3389/fneur.2025.1584226

**Published:** 2025-07-07

**Authors:** Xiaoyan Zhou, Haoyong Zhu, Xiaoming Wang, Qing Gao

**Affiliations:** ^1^First Clinical Medical College, Jinan university, Guangzhou, China; ^2^Department of Neurology, Affiliated Hospital of North Sichuan Medical College, Nanchong, China; ^3^Health Management Center, Affiliated Hospital of North Sichuan Medical College, Nanchong, China; ^4^The Clinical Hospital of Chengdu Brain Science Institute, MOE Key Laboratory for Neuroinformation, High-Field Magnetic Resonance Brain Imaging Key Laboratory of Sichuan Province, School of Mathematical Sciences, University of Electronic Science and Technology of China, Chengdu, China

**Keywords:** machine learning, structure MRI, brain age, Parkinson’s disease, shapley additive explanations

## Abstract

**Introduction:**

This study explores the use of imaging to evaluate brain aging to establish a model for predicting brain age in patients with Parkinson’s disease.

**Methods:**

Structural brain MRI data from 345 healthy individuals were obtained from the IXI database, while data from 59 Parkinson’s patients and 59 healthy controls were acquired from the PPMI database. A total of 1214 structural indicators were extracted, including information on the whole brain, cortex, subcortex, and white matter. This led to the development of a model for predicting brain age in Parkinson’s patients. The model combined brain imaging features with a machine learning algorithm and the Shapley Additive Explanations (SHAP) interpretation model. Fifteen characteristic indicators most closely associated with Parkinson’s brain aging were determined.

**Results:**

The XGBoost model + SHAP method framework, using the minimum mean absolute error for assessing brain aging within 4.21 years, was effective in predicting brain age in patients with Parkinson’s disease. The superior temporal folding index and subcortical gray matter volume, left thalamus volume, and left and right vascular volumes had the most significant impact on the prediction results, suggesting their potential as clinical indicators for evaluating the extent of brain aging in Parkinson’s patients.

**Discussion:**

These findings provide important clues for understanding the mechanisms underlying brain aging, as well as brain imaging evidence for the early diagnosis and treatment of Parkinson’s disease.

## Introduction

Parkinson’s disease (PD), a common neurodegenerative disorder, is pathologically characterized by neuronal loss and structural remodeling in specific regions of the brain (1, 2), leading to motor dysfunction with typical clinical symptoms such as tremor, muscle rigidity, bradykinesia, and postural balance disorders (3, 4). Normal brain aging is a natural progessive physiological process, while PD deviates from this process by damaging specific neuronal populations in the brain, resulting in a characteristic pathological aging pattern (5). With the rapid development of neuroimaging techniques, functional imaging has become an important tool for the diagnosis and monitoring of diseases of the nervous system. Machine-learning models based on a quantitative analysis of structural changes in brain regions can be used to estimate an individual’s brain age. The difference between this estimated value and the actual physiological age is defined as the brain age gap (BAG), i.e., predicted brain age - actual age (6). The BAG value can provide a reflection of changes associated with abnormal brain aging, and can be used for the early diagnosis of age-related neurodegenerative diseases (7–9). A large-scale analysis of 11,000 patients with various neuropsychiatric disorders suggested that a BAG value indicative of increased brain age may be a sensitive, although possibly not specific, biomarker (10). A study based on the ADNI dataset, using an automatic brain age estimator to analyze clinical samples from healthy individuals (HC) and patients with Alzheimer’s disease (AD) showed that the average BAG in estimating brain age was 4.98 years in the HC group, while the value in the AD group based on gray matter (GM) features was 10 years (indicating that the predicted brain age was significantly higher than the actual age), the study further verified the value of using BAG in the early diagnosis of AD, as well as in predicting the conversion of mild cognitive impairment (MCI) to AD (11). However, there are few studies on specific brain age models for PD, and utilization of artificial intelligence is insufficient. In this context, the present study used a machine-learning model to predict brain age in PD patients in the dataset, aiming to explore the relationship between BAG and PD-related changes in brain structure. We hypothesized that patients with PD exhibit significantly elevated BAG values compared to age-matched controls, especially in brain regions known to be affected during early stages of the disease. To a certain extent, this can reveal the underlying pathogenesis of Parkinson’s disease and structural changes within the brain throughout the course of the disease, which would be helpful for both the early diagnosis of Parkinson’s disease and assessment of its disease course.

## Methods

### Data sources

The information extraction from images (IXI) brain dataset, created by the London Health Sciences Centre at University College London, UK, is widely used for neuroscience research and medical imaging. The IXI dataset contains a wealth of neuroimaging data, including structural magnetic resonance imaging, functional MRI (fMRI), and brain magnetic resonance spectroscopy (MRS) data.

Specifically, the IXI Brain dataset provides information in various key areas. This information represents, firstly, structural MRI images, including high-resolution T1-and T2-weighted imaging. These images provide detailed information on brain anatomy and are useful for investigations into the segmentation of brain regions and brain structure. Secondly, functional MRI data on spatial and temporal brain activities during different task states; these data are usually obtained by measuring cerebral blood flow or oxygenation levels, and provide an important foundation for the evaluation of brain functional connectivity and activity patterns. The last category is MRS data, which provides information on the levels of various metabolites, such as creatine, choline, and pyruvate, in the brain tissue. These data are important for determining the relationship between the metabolic state of the brain and neurological disorders.

The release of the IXI Brain dataset has had a significant impact on neuroimaging research and medical diagnosis. The dataset provides researchers and physicians with a wealth of brain imaging data, enabling a deeper understanding of the relationships between brain structure and function and the associations between brain imaging features and disease. At the same time, the dataset also provides a valuable resource for research in the fields of machine learning and artificial intelligence, driving the continuous development of automated neuroimaging-based analysis and diagnostic techniques. In the present study, the data of 345 healthy individuals were obtained from the IXI, while information on 118 individuals was acquired from the Parkinson’s Progression Markers Initiative (PPMI) dataset. The PPMI dataset is an open-access research resource designed to facilitate a deeper understanding of the pathological progression of PD through the collection of extensive clinical, imaging, genomic, and biomarker-related data. The PPMI initiative, launched by the Michael J. Fox Foundation for Parkinson’s Research, was established to identify early biomarkers of PD and promote the development of novel therapeutic approaches. The latter included data from 59 healthy individuals and 59 patients with PD, with the healthy group and the PD group controlled against each other. The IXI data were used as the training set and the PPMI data as the test set, and a machine learning model was applied to predict brain age. This study used the Unified Parkinson’s Disease Rating Scale (UPDRS) and the Montreal Cognitive Assessment (MoCA) scores to, respectively, assess PD severity and cognitive impairment in the PD group. The demographic information of the study participants is summarized in Table 1. No significant differences were observed between the PD and HC cohorts in terms of age (*t*, *p* = 0.533) and sex distribution (*χ*^2^, *p* = 0.102).

**Table 1:** Information on study subjects.

Information on study subjects	Training set	PD group	Healthy group *p*-value
Average age (in years)	44	61	62 0.533^c^
Sex ratio (men to women)	146: 199	22:37	30:29 0.102^d^
UPDRS^a^ (mean)	–	20	–
MoCA^b^ (mean)	–	27	–

^a^Unified Parkinson’s Disease Rating Scale score, ^b^Montreal Cognitive Assessment score, ^c^T test, ^d^χ2 test.

#### Subject inclusion and exclusion criteria

Potential participants from the IXI dataset were included in the analysis if they met all of the following criteria: (1) availability of T1-weighted MRI scans, (2) aged between 20 and 80 years, and (3) no documented major neurological or psychiatric disorders based on metadata. The exclusion criteria comprised: (1) poor quality of scans, determined by visual inspection/QC reports; (2) incomplete demographic information; or (3) the presence of significant motion artifacts.

Patients with *de novo* PD from the Parkinson’s Progression Markers Initiative (PPMI) cohort were included if they satisfied the following criteria: (1) the availability of high-quality T1-weighted MRI scans; and (2) aged between 20 and 80 years. The exclusion criteria included: (1) diagnosis of non-idiopathic PD; (2) poor scan quality identified by PPMI quality control or our visual assessment; or (3) comorbid neurological conditions documented in the PPMI records.

Healthy controls (HC) from the PPMI dataset were included with 1:1 matching to PD participants based on the following criteria: (1) availability of T1-weighted MRI scans; (2) aged between 20 and 80 years; and (3) an absence of major neurological/psychiatric disorders. The exclusion criteria were the same as those used for the IXI participants.

#### Data acquisition

T1-weighted MRI scans from the IXI and PPMI datasets were acquired at consistent 3 T field strength using comparable MPRAGE sequences. The key acquisition parameters exhibited high compatibility, including the repetition time (TR: IXI = 7.5–9.5 ms, PPMI = 7.8 ms, *Δ* < 3%), echo time (TE: IXI = 3.5–4.5 ms, PPMI = 3.7 ms, Δ < 5%), and flip angle (FA = 8° identical). Minor variations in spatial resolution (IXI = 0.94 × 0.94 × 1.2 mm^3^, PPMI = 1.0^3^ mm^3^) and slice thickness (IXI = 1.2 mm, PPMI = 1.0 mm) were harmonized by spatial normalization to the MNI152 1 mm isotropic space using ANTs SyN registration. Differences in scanner manufacturers (IXI: Siemens/Philips; PPMI: Siemens-dominant) were controlled statistically as categorical covariates in all group analyses. This preprocessing pipeline ensured consistency of all parameters, enabling cross-dataset comparisons.

#### Data pre-processing

**Figure 1:**
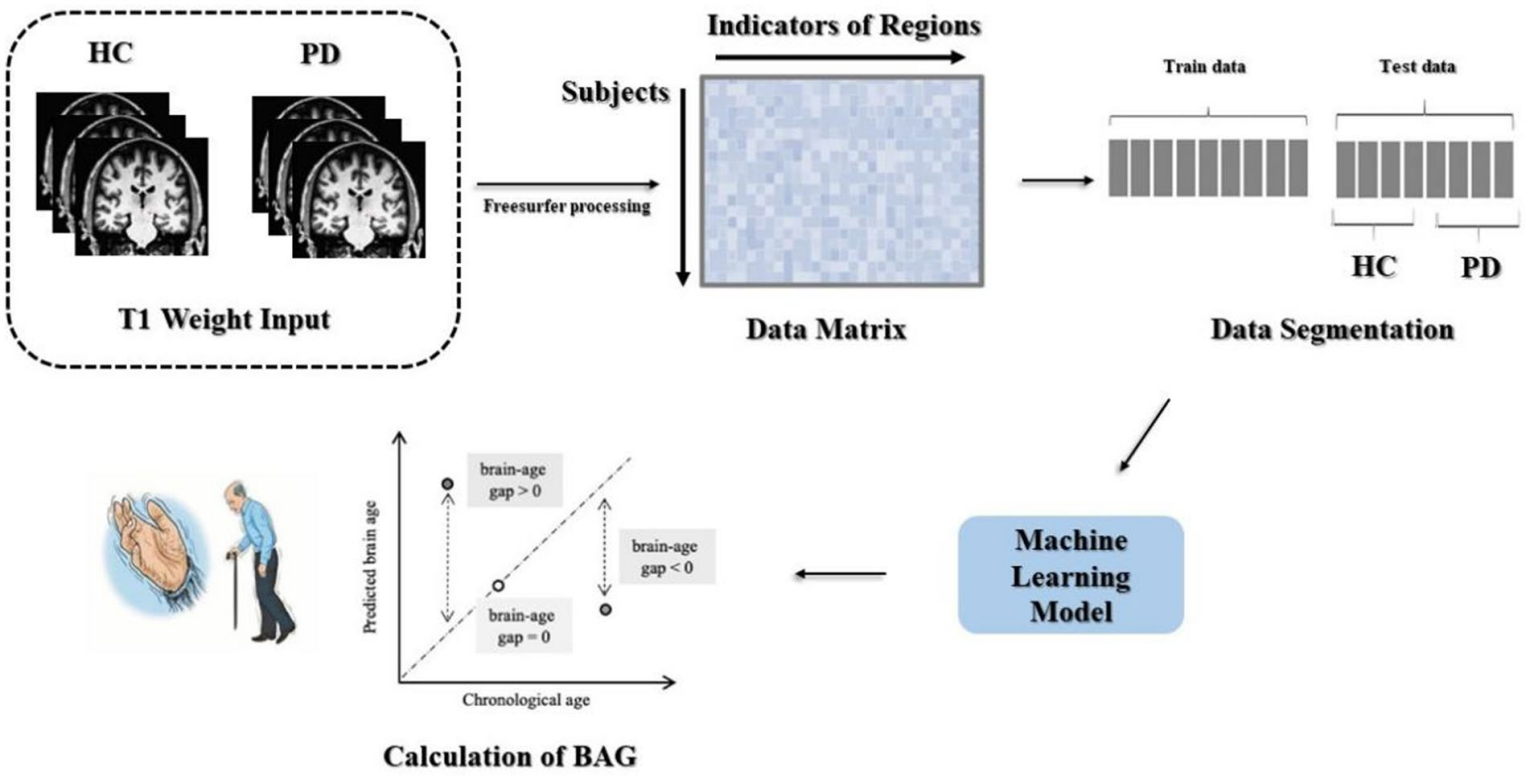
Data preprocessing and model building.

Processing of the data included head movement correction, non-uniform intensity normalization, cortical band masking, and cortical partition mapping, with an overall 31 steps. The output files contained statistical indices such as cortical thickness, curvature, and volume, in addition to the operational log files. This assisted the researchers in identifying issues during the execution process, making it easier to control the quality of the final data. The steps involved in data processing are depicted in Figure 1.

The generated log files were reviewed to ensure that the data processing was complete and correct at the end of the run. The data included a total of 1,214 brain metrics, such as 40 subcortical brain regions templated with the ASEG template and 68 statistical metrics of brain regions of interest (ROI) of the Desikan-Killiany subcortical region template, as well as white matter and whole-brain metrics. These included: firstly, volume, intensity mean, standard deviation, minimum, maximum, and range for 40 subcortical brain structures and white matter subdivisions of the cerebral cortex; secondly, volume, surface area, Gaussian curvature, mean curvature, curvature index, folding index, thickness mean, and thickness standard deviation for 34 cortical brain regions in each hemisphere; thirdly, whole-brain metrics, including surface area and volume statistics for each hemisphere, total cerebellar gray and white matter volumes, brainstem volume, and corpus callosum volumes. Specifically, there were four categories defined by the type of metric, namely, 10 metrics for whole-brain, cortical metrics (544 features derived from 8 metrics for 68 cortical ROIs), subcortical metrics (240 features derived from 6 metrics for 40 subcortical ROIs), and white matter metrics of 420 features generated from 6 metrics for 70 ROIs.

#### Machine learning pipeline

The data underwent preprocessing to yield two matrices with respective sizes of 345 × 1,214 and 118 × 1,214. Using the age vectors of the test and training sets from the participant information as the prediction targets, the MAE and correlation coefficient could be computed to assess the goodness-of-fit of the model. The trained machine-learning model was then applied to the test set to evaluate the overall performance of the model. The method used for calculating the BAG is shown in Figure 1.

#### Evaluation metrics

Brain age prediction is a regression problem, and supervised machine learning algorithms are effective when using datasets of a certain size. The evaluation metric for brain age prediction was the mean absolute error (MAE). If $y_{i}$ is the true age, $\hat{\;y_{i}}$ is the predicted age, $\;\bar{\hat{y_{i}}}$ is the mean of the predicted age, $\;\bar{y}$ is the mean of the true age, and N is the sample size, the formula for calculating the MAE is:

$$\mathrm{MAE}=\frac{1}{N}\sum_{i=1}^{N}|y_{i}-\hat{y_{i}}|$$

The Pearson correlation coefficient between the predicted and actual age R can then be calculated as:

$$R=\frac{\sum_{i=1}^{N}(y_{i}-\bar{y})(\hat{y_{i}}-\bar{\hat{y_{i}}})}{\sqrt{\sum_{i=1}^{N}{\left(y_{i}-\bar{y}\right)}^{2}}\sqrt{\sum_{i=1}^{N}{\left(\hat{y_{i}}-\bar{\hat{y_{i}}}\right)}^{2}}}$$

#### BAG calculation

The BAG is a biomarker representing the difference between the aging trajectory of an individual brain and that of the normal group, with greater BAG values implying accelerated brain aging relative to normal individual aging. In this study, the test set was divided into healthy and PD groups, and calculation of the BAG between the two groups enabled the determination of the presence or absence of accelerated aging in PD patients. It is also possible to add covariates to the analysis, such as developmental years, duration of the disease, Hamilton Depression Rating Scale (HDRS) scores, Montreal Cognitive Assessment Scale (MoCA) scores, and Unified Parkinson’s Disease Rating Scale (UPDRS) scores, to identify factors that might influence brain aging (12). As described in this paper, this enables the identification of regional brain indicators that affect the prediction of brain aging, thus allowing a more accurate assessment of brain age.

## Results

### Age correction

A systematic bias was observed in the predicted ages of subjects of all ages, indicating an over-prediction of age in relatively younger individuals and an under-prediction in older individuals. The underlying cause of this bias remains obscure in general nonlinear prediction methods. Le et al. showed that this bias is inevitable in regression, rather than being a property limited to age prediction (13). It has been defined as ‘regression dilution’, attributed to the non-Gaussian distribution of chronological age. Zhang et al. have summarized methods used for age correction (14), and the present study used a sample-level age correction method to address this bias.

**Figure 2:**
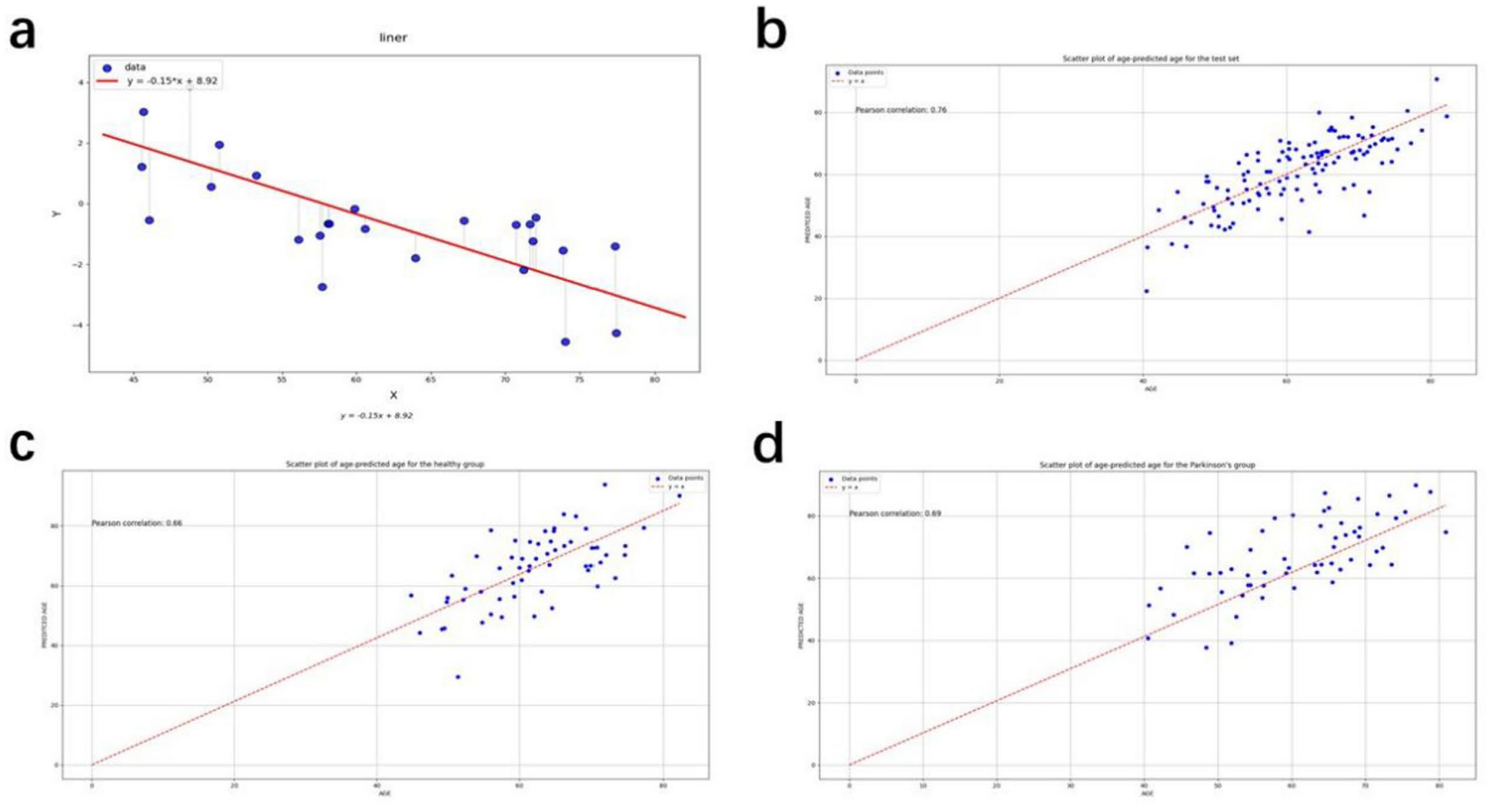
**(a)** Straight line fitted to the gap between true age and brain age for the independent set; **(b)** Scatter plot of age-predicted age for the test set; **(c)** Scatter plot of age-predicted age for the healthy group; **(d)** Scatter plot of age-predicted age for the Parkinson’s group.

The calibration procedure was designed to cope with the dependence of the prediction process, i.e., all predicted individuals would be close to the trend of the overall average, which would lead to a certain amount of bias. This would increase the difficulty of interpretation in both the control and patient sets. The slope α and the intercept β were calculated by fitting a straight line between the true age and the BAG. The OFFset was calculated by this process. The true age was represented by Ω , and the predicted age of the test set was subtracted from the offset to obtain the corrected predicted age (Figure 2a).

OFFset=αΩ+β

α was calculated to be −0.02, and β was calculated to be 0.7. Both the brain age gap and the mean absolute error, obtained below, were derived on corrected ages. The parameters here are calculated based on an independent set. The independent data set is the PPMI healthy control test set, and the age distribution of the data is approximately the same as that of the PD group and HC group in the test set. There are a total of 25 participants.

### Performance of the regression model

Four regression models for the prediction of brain age are described in this paper, and we used the optuna Bayesian optimization tuning algorithm. The results of the recursive feature elimination method were used for comparison with the unselected features, it was found that the accuracy of the model in which features had been eliminated was decreased to a certain extent compared with the model containing all the features, and thus feature selection was discontinued. Parameter optimization of the model was performed. The model was trained several times. The 95% confidence intervals for the MAE and R before and after calibration were obtained using the bootstrap method, as shown in Table 2.

**Table 2:** Model performance.

Model	MAE^e^	R^e^	MAE^f^	R^f^
XGboost^a^	4.21 ± 0.19	0.76 ± 0.03	5.80 ± 0.38	0.60 ± 0.03
RF^b^	6.45 ± 0.26	0.58 ± 0.05	7.10 ± 0.42	0.45 ± 0.05
SVR^c^	6.77 ± 0.23	0.55 ± 0.03	7.50 ± 0.45	0.40 ± 0.04
LASSO^d^	6.21 ± 0.18	0.61 ± 0.02	6.60 ± 0.40	0.50 ± 0.03

^a^eXtreme Gradient Boosting. ^b^Random Forest. ^c^Support Vector Regression.

^d^Least Absolute Shrinkage and Selection Operator.

^e^After correction.

^f^Before correction.

XGBoost was found to be most suitable for analysis of the present structured heterogeneous tabular dataset, enabling the integration of clinical metrics and neuroimaging features. Its gradient-boosted tree architecture can efficiently capture complex interactions between features and nonlinear relationships inherent to biomedical data. Compared to conventional Gradient Boosted Decision Trees (GBDT), XGBoost incorporates advanced regularization techniques (L1/L2 penalties on leaf weights and complexity control) and sophisticated tree-pruning methodologies. These innovations significantly mitigate overfitting, a critical advantage given our limited sample size. This robustness is in sharp contrast with deep learning models, which typically require substantially larger datasets to achieve comparable generalization performance. While Random Forest (RF) models can also handle tabular data effectively and provide interpretability, XGBoost can achieve a consistently higher prediction accuracy under an equivalent tuning effort, while exhibiting stronger resistance to overfitting in small-sample scenarios. Deep neural networks were considered suboptimal due to three primary limitations, specifically, their increased risk of overfitting in smaller samples, their computational complexity potentially exceeding practical constraints, and inadequate interpretability for clinical deployment.

Hyperparameter optimization employed a grid search across critical parameters: max_depth, n_estimators, learning_rate, min_child_weight, gamma, subsample.

The XGBoost model was ultimately found to yield the best results, and was thus chosen as the model for subsequent study. The corrected metrics were computed using this model, and the average absolute error of the corrected metrics over the entire test set was found to be 4.21 years. This is an improvement in accuracy compared to the uncorrected model. The model was used for prediction to derive a predicted age, which was then corrected using the correction procedure. Scatter plots of the true age and the corrected predicted age were compiled, as shown in Figures 2b–d. The red lines in the three figures represent the predicted ages for the test set, healthy group, and PD group, defined by the baseline y = x. The points on the baseline indicate that the predicted age equals the true age. The closer the points are to the baseline, the better the prediction. The points in Figures 2b–d are seen to cluster near the baseline, indicating the accuracy of the prediction results. The Pearson correlation coefficients are 0.671, 0.716, and 0.653, respectively, demonstrating the effectiveness of the prediction model.

### Model interpretation results

**Figure 3:**
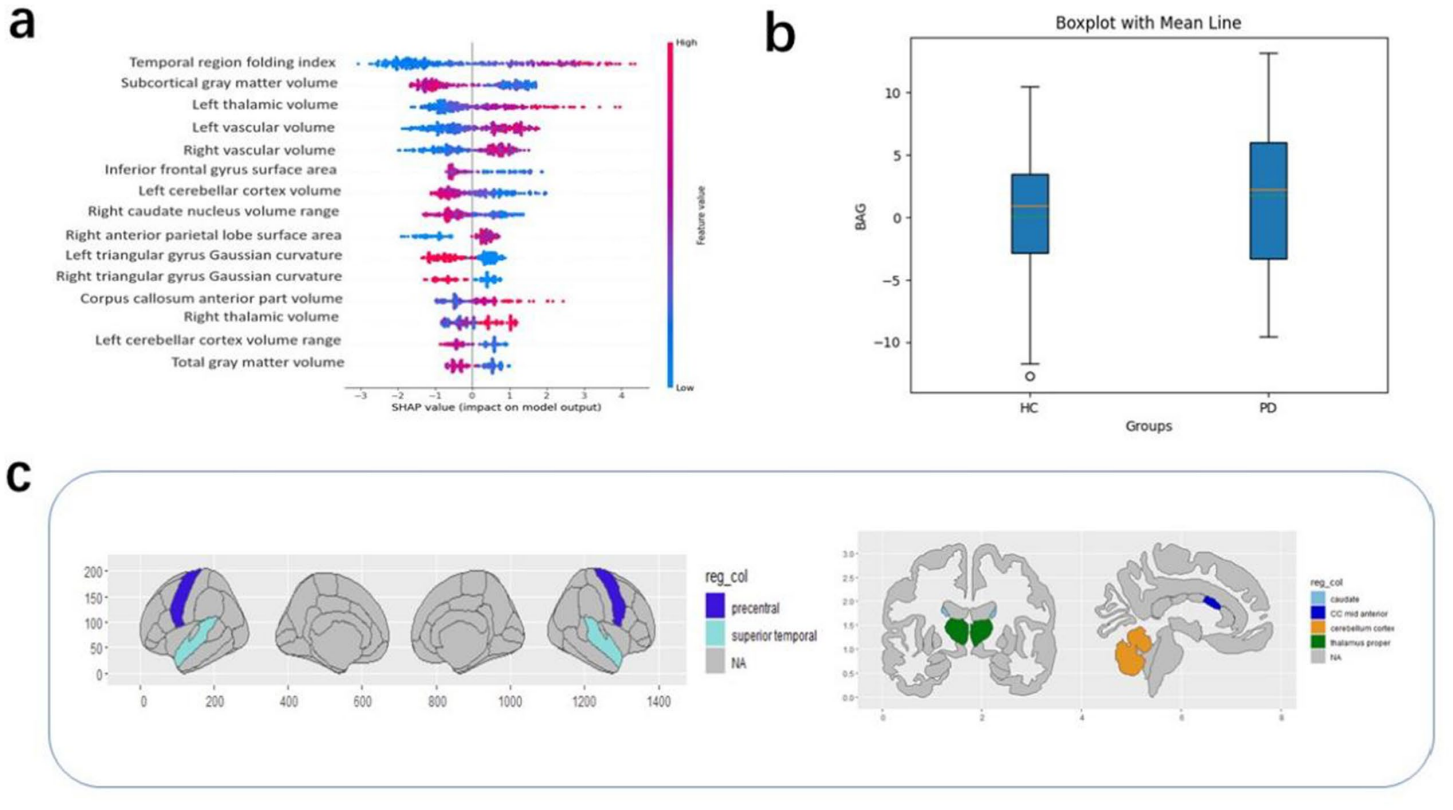
**(a)** The top 15 features in order of importance; **(b)** BAG values according to groups in the test set. **(c)** High SHAP values correspond to regions of interest.

The purpose of the SHAP interpretation method is to generate an N × M matrix, where N represents the number of individuals and M is the number of individual characteristics. An individual value in the SHAP matrix represents the average contribution of a particular feature to the predicted age for a given individual. This can reflect the importance of the feature to some extent. The SHAP method was used to generate an interpretation matrix in the XGBoost regression model. Figure 3a shows the SHAP values and the 15 most important features. This result is based on the mean SHAP values. The corrected predicted age was then used to calculate the BAG, i.e., predicted age minus true age. A value greater than 0 indicates that the brain appears “older” than normal, while a value less than 0 indicates that the brain appears younger than normal. Figure 3b shows that the mean BAG was greater than 0 for both groups in the test set, with the PD group having a mean BAG of 1.79 years, while the mean BAG in the healthy group was 0.08 years. This indicates the presence of accelerated aging in the PD group compared to the healthy group.

Other covariates in the PD group dataset, such as the patients’ years of education, duration of illness, HDRS scores, MoCA scores, and UPDRS scores, were analyzed in relation to the BAG. These covariates did not significantly affect the BAG. Therefore, there was no good reason to suggest a correlation between them and the brain age gap (15). To understand which brain-based features contributed to the BAG (regardless of diagnosis), the SHAP value was calculated for each of the 1,214 features and for each participant, followed by averaging of this absolute value for all participants in each dataset (regardless of group). The 15 most relevant features based on their average absolute contribution to the prediction were extracted from the PD group in the test set and these features were then analyzed for each dataset (Figure 3). In descending order of feature importance, these were the superior temporal folding index, subcortical gray matter volume, left thalamic volume, left vascular volume, right vascular volume, inferior frontal gyrus surface area, left cerebellar cortex volume maxima, right caudate nucleus volume range, right anterior parietal surface area, left deltoid gyrus Gaussian curvature, right deltoid gyrus Gaussian curvature, mid anterior corpus callosum volume maxima, right thalamocortical volume maximum, left cerebellar cortex volume range, and total gray matter volume.

## Discussion

The BAG value provides an objective reflection of abnormal changes in the aging characteristics of the brain, enabling the analysis of disease risks and causative factors. Consequently, the use of the BAG as a reliable biomarker has attracted significant attention in the field of brain diseases. Notably, higher BAG values are associated with an elevated risk of numerous neuropsychiatric disorders, including AD, PD, Multiple Sclerosis (MS), and schizophrenia. Additionally, extensive research has demonstrated that certain neuropsychiatric conditions can alter the brain structure and expedite brain aging, resulting in a brain age above that of the actual chronological age (8, 17–19). Previous studies have shown that for all investigated models, AD and MS exhibited the most pronounced BAG values (20, 21). Specifically, the brain ages of patients with these diseases were found to be over 10 years higher than their actual chronological ages, and were significantly correlated with scores of cognitive ability (such as MMSE) and dysfunction (such as EDSS) (22). Liu et al. reported that for every annual increment in the BAG, the risk of developing AD was increased by 4.57% (23). Moreover, individuals with MCI and a higher BAG value were also at a heightened risk of progressing to AD (24). This implies that brain age, as indicated by the BAG, can predict the likelihood of progression to serious neurodegenerative conditions and plays a crucial part in both predicting and differentiating AD from MCI (25). Cole et al. reported that a higher BAG value was associated with poorer raw cognitive abilities, thereby highlighting the significant potential of the BAG in identifying cognitive impairment (22). Another comparative study focusing on BAG values in PD and AD patients revealed that the brains of AD patients might be considerably “older” than those of PD patients (26). As an imaging biomarker, the BAG can, to a certain degree, reflect the relevant alterations occurring within the brain and provide an early predictor of disease diagnosis and severity.

In the present study, a machine learning model was employed to forecast the brain age of individuals, using data from a dataset of brain region metrics derived from structural magnetic resonance images. After adjustment for the predicted age, the BAG was computed, and was shown to be effective for assessing accelerated brain aging in patients with PD. Further analysis of the BAG in PD patients indicated that, in the test set, the mean BAG value was greater than zero in both groups. Specifically, the PD group exhibited a mean BAG value of 1.79 years, in contrast to a value of 0.08 years in the healthy group. This indicated the presence of accelerated aging in the PD group relative to the healthy cohort; these findings are consistent with those of previous studies on brain age (26). As the disease advances, the brain ages at an expedited pace, accompanied by corresponding alterations in anatomical structures. These changes, in turn, lead to reduced brain volume and function, and manifest as increased BAG values. Additionally, we examined the correlations between BAG values and other covariates in the PD group, including patients’ years of education, disease duration, HDRS scores, MoCA scores, and UPDRS scores. Intriguingly, it was found that the increased brain age in PD patients was not associated with the particular clinical characteristics evaluated in this study. This suggests that, in contrast to an individual’s underlying physiological status, encompassing genetic background, health, and lifestyle habits, factors such as educational level, illness duration, and clinical symptoms of PD patients might have only a relatively minor influence on brain age. Brain aging likely follows a strongly conserved molecular regulatory mechanism. A comprehensive meta-analysis of genes expressed in the human prefrontal cortex from multiple databases showed that the most marked characteristic of the aging process in this brain region was downregulation of genes associated with synaptic transmission. Reduced expression of genes related to neural regeneration was also common (27). The mitochondria, which represent the energy hubs of cells, play a pivotal role in the electrophysiological activities of neurons as well as in synaptic plasticity. Studies have demonstrated that reduced expression of genes involved in mitochondrial energy metabolism is particularly pronounced in individuals with cognitive dysfunction (28, 29). Moreover, a substantial body of research evidence suggests that lifestyle interventions, including dietary modification, physical exercise, and cognitive training, have a positive impact on retarding the brain aging process (30, 31). In this study, the absence of a correlation between the clinical scale and the BAG might also be attributed toa lack of sensitivity in the clinical scales used for detection of subclinical neurodegeneration. However, these presumptions necessitate further exploration in subsequent studies to disentangle the impact of lifestyle characteristics from raw scores and reconcile the relationships between physiological and pathological patterns. Such efforts are crucial for the identification of sensitive, specific, and clinically valuable biomarkers that can enhance our understanding of patient heterogeneity.

In addition, within the scope of our study, several brain regions were found to make more substantial contributions to the outcome of the brain age prediction. These included the superior temporal folding index, subcortical gray matter volume, left thalamic volume, left vascular volume, and right vascular volume. Eickhoff et al. reported an association between increased brain age and atrophy in diverse brain regions. In particular, patients with PD showed extensive cortical atrophy, most notably in the right central region and medial frontal lobe, as well as in the visual and temporal cortices and the thalamus and basal ganglia. Notably, a negative correlation was observed between brain age and gray matter volume (32). A meta-analysis further demonstrated significantly reduced gray matter volumes in specific brain regions in PD patients (33). In this study, significant differences were observed in the volumes of the thalamus and bilateral blood vessels between the PD and control groups. This is potentially attributable to the neuronal loss, specifically the progressive death of dopaminergic neurons, characteristic of PD. The thalamic and vascular regions of the brain are linked directly to dopaminergic neurons. In the early stages of the disease, as the number of dopaminergic neurons declines, the brain structures endeavor to compensate, manifesting as increases in the thalamic and vascular volumes. However, with further disease progression and the continued death of dopaminergic neurons, the thalamic and vascular volumes may decrease due to the loss of their compensatory capacity. Consequently, the atrophy induced by PD, which differs from normal age-related degeneration, might lead to a more pronounced BAG and prove instrumental in the clinical diagnosis of early PD.

In terms of model accuracy, the mean absolute error of the corrected XGBoost model was calculated to be 4.21 years. This figure deviates somewhat from the optimal outcome of 2.90 years achieved in the prediction contest organized by Predictive Analytics Competition (PAC). The probable cause for this disparity lies in the fact that the dataset utilized in our study encompassed only 284 samples, which is considerably smaller than that of PAC. The relatively small sample size may affect the generalizability of the findings. However, the reason we chose XGBoost is that it offers its superior interpretability and capacity to model nonlinear relationships, despite marginally higher mean absolute error (MAE) compared to deep learning architectures. XGBoost provides transparent feature importance quantification, enhancing model trustworthiness through explicit visualization of prediction drivers. Its gradient-boosting framework effectively captures complex nonlinear interactions while ensuring robustness across heterogeneous biomedical datasets. Integration with SHAP further augments model transparency by enabling granular interpretation of feature contributions at both global and individual prediction levels.

In summary, the findings of this study strongly indicate that the BAG, serving as a stable and dependable imaging biomarker, has significant potential in the evaluation of structural changes in the brains of patients with PD. The use of the BAG can provide clinicians with valuable insights that enable both the early identification of patients and the implementation of stratified management strategies. Future investigations should also further dissect the significance of different clinical subtypes or characteristics of PD and the use of individualized predictions of brain age in the early diagnosis of the disease.

## Limitation

This study included data from multiple sites, which may potentially have introduced heterogeneity, particularly in terms of multi-site effects and differences in scanners. Factors such as experimental conditions, equipment configurations, operators, and scanner models can influence the data, potentially leading to biases during acquisition and processing (34). For instance, differences in scanner hardware can impact image quality, while variations in experimental conditions may lead to data inconsistencies (35). Such heterogeneity could affect the generalizability and reliability of the study’s conclusions. It is important to note that no data harmonization techniques, such as ComBat, were used to address biases from different sites and scanners. ComBat has been shown to effectively correct batch effects in multi-site data, enhancing the stability of the results (36). In the absence of these techniques, errors caused by heterogeneity of data sources may have influenced the accuracy of the conclusions. Future investigations could mitigate these biases by using data harmonization methods, improving the reproducibility and reliability of the results. Additionally, the sample size was relatively small. Despite its collection from multiple sites, the sample may not fully capture various sources of heterogeneity. Smaller datasets are more susceptible to noise, limiting model generalizability. Insufficient sample sizes in multi-site studies can also lead to difficulties in the detection of site-specific differences, affecting the accuracy of the analysis. Future studies should consider increasing the sample size and performing further investigations of heterogeneity in data from multiple sites. Finally, the analytical methods may not have fully accounted for site and device effects. While standardized preprocessing reduces the likelihood of systematic errors, specialized correction methods are needed to address differences in devices and sites. Future research could refine the preprocessing steps, including the use of device calibration and site effect modeling, to enhance the reliability of the results. In conclusion, data heterogeneity, multi-site effects, and the lack of harmonization techniques are key study limitations. Future research should apply data harmonization methods with larger sample sizes to improve accuracy and generalizability. The relationship between covariates and BAG was analyzed using methods such as regression. However, the hypothesis test failed to pass. Considering the sample size and the influence of errors, control of both data quality and errors will be the focus of our next steps.

## Data Availability

The raw data supporting the conclusions of this article will be made available by the authors, without undue reservation.
