# Correction: A predictive model of Parkinsonian brain aging based on brain imaging features

**DOI:** 10.3389/fneur.2025.1712929

**Published:** 2025-10-07

**Authors:** Xiaoyan Zhou, Haoyong Zhu, Xiaoming Wang, Qing Gao

**Affiliations:** ^1^First Clinical Medical College, Jinan University, Guangzhou, China; ^2^Department of Neurology, Affiliated Hospital of North Sichuan Medical College, Nanchong, China; ^3^Health Management Center, Affiliated Hospital of North Sichuan Medical College, Nanchong, China; ^4^The Clinical Hospital of Chengdu Brain Science Institute, MOE Key Laboratory for Neuroinformation, High-Field Magnetic Resonance Brain Imaging Key Laboratory of Sichuan Province, School of Mathematical Sciences, University of Electronic Science and Technology of China, Chengdu, China

**Keywords:** machine learning, structure MRI, brain age, Parkinson's disease, shapley additive explanations

In the published article, the statement “These authors have contributed equally to this work” was erroneously not added for authors Xiaoyan Zhou and Haoyong Zhu.

The original version of this article has been updated.

